# High prevalence of osteoporosis in patients undergoing spine surgery in China

**DOI:** 10.1186/s12877-021-02313-8

**Published:** 2021-06-13

**Authors:** Xiaoyi Mo, Shengli Zhao, Zhenxing Wen, Wei Lin, Zhipeng Chen, Zhiyun Wang, Chen Huang, Jie Qin, Jie Hao, Bailing Chen

**Affiliations:** 1 Department of Spine Surgery, The First Affiliated Hospital of Sun Yat-sen University, Guangzhou, China; 2 Guangdong Provincial Key Laboratory of Orthopaedics and Traumatology, Guangzhou, China; 3 Department of Spine Surgery, Shunde Hospital of Southern Medical University, Foshan, China; 4 Department of Orthopaedic, Yantai Mountain Hospital, Yantai, Shandong China; 5 Department of Spine Surgery, The First Affiliated Hospital of Chongqing Medical University, Chongqing, China

**Keywords:** Osteoporosis, Prevalence, Spine surgery, Vertebral fracture, Chinese

## Abstract

**Background:**

With the increase in life expectancy, a large number of patients with osteoporosis (OP) are undergoing spine surgery, which may adversely affect the surgical success rate. The prevalence of OP varies in different regions, and no data are available that represent the prevalence of OP among Chinese patients over 50 years of age who are undergoing spine surgery. It was the first multicenter study to assess OP in these patients. Aiming to obtain comprehensive data, this study combined bone mineral density (BMD) measurements and visual radiography assessment (VRA) to analyze the prevalence of OP in patients aged > 50 years who underwent spine surgery.

**Methods:**

Data from 1,856 patients aged over 50 years undergoing spine surgery who resided in northern, central, and southern China were reviewed between 2018 and 2019. Based on the perioperative BMD and X-ray data, we calculated the prevalence of OP in this special population according to sex, age, and spine degenerative disease.

**Results:**

A total of 1,245 patients (678 females and 567 males) were included in the study. The prevalence of OP diagnosed by BMD was 52.8 % in females and 18.7 % in males. When we combined with BMD and VRA, the prevalence of OP increased from 52.8 to 65.9 % in females and from 18.7 to 40.6 % in males. Although OP was more severe in females than in males, a significant difference in the rate of vertebral fracture (VF) was not observed between females and males with a normal BMD and osteopenia (females vs. males: aged 50–59 years, *P* = 0.977; 60–69 years, *P* = 0.302; >70 years, *P* = 0.172). Similarly, no significant difference in the vertebral fracture rate was observed within different age groups of patients with a normal BMD and osteopenia (females: *P* = 0.210; males, *P* = 0.895). The incidence of OP in patients with degenerative scoliosis was higher than that in the remaining patients (females: 63.6 % vs. 42.4 %, *P* = 0.018; males: 38.9 % vs. 13.8 %, *P* = 0.004).

**Conclusions:**

A high prevalence of OP was identified in patients aged > 50 years undergoing spine surgery, especially in patients whose primary diagnosis was degenerative scoliosis. BMD and VRA evaluations should be included in the clinical routine for these patients prior to surgery.

## Background

Osteoporosis (OP) is a metabolic disease characterized by a low bone mineral density (BMD) and the microarchitectural deterioration of bone tissue leading to fragility fractures. Primary OP is extremely common in the elderly. China has both the largest elderly population and the fastest rate of increase in the elderly population worldwide. Currently, the proportion of the elderly population in China is 15.5 % and will increase to 31.2 % by 2050 [1]. According to a recent nationwide and multicenter survey in China, the prevalence of OP in females and males aged > 50 years was 29.13 and 6.46 %, respectively [2], which would reach 39.19 and 7.46 %, respectively, by 2050 [1]. Vertebral fracture (VF) is a potentially severe complication of OP because of protracted back pain, impaired quality of life, and increased disability [3]. The annual number of OP-related fractures is predicted to reach 4.83 million by 2035 in China, which will cost approximately $19.92 billion [4].

With population aging, spine surgeons must manage many elderly patients with a low bone quality because of OP, and this number will continue to increase as the baby boomer generation ages. A large number of studies have shown that a low BMD in elderly patients may lead to several complications, such as instrumentation loosening, adjacent segment fractures, and lower fusion rates [5–7]. An AOSpine Latin America survey reported that approximately 71 % of spine surgeons had revised their instrumentation because of OP-related complications [8]. Thus, spine surgeons must be alerted to the severe situation of OP, which will allow spine surgeons to be well prepared during the perioperative period.

Dual-energy X-ray absorptiometry (DXA) is recommended as the gold standard method for defining OP by the World Health Organization [9], and the most broadly recognized site for DXA is the axial skeleton (lumbar spine (L1-4)) and the hips (femoral neck and total hip) [10]. Due to the effect of lumbar degenerative changes, abdominal aortic calcification, and hip osteoarthritis, the BMD may be falsely increased [11, 12], and thus many elderly patients with VF do not have a T-score consistent with an OP diagnosis. As a supplement to BMD, conventional radiography is presumed to be the best method for the detection of VFs. Every patient undergoing spine surgery undergoes a routine chest X-ray and a spinal X-ray of the segment requiring surgery, which can be used to evaluate the VFs based on the method of visual radiography assessment (VRA). This approach not only helps to detect undiagnosed OP but also decreases the cost and provides convenience to patients.

In the present study, we hypothesized that combining DXA and VRA would reveal a high prevalence of OP in patients undergoing spine surgery in China and that a large number of these patients would have been undiagnosed previously.

## Methods

### Participants

 From September 1, 2018, to December 31, 2019, 1,856 patients treated at four different medical centers in three regions (two centers from Guangdong Province, *n* = 669; one center from Chongqing municipality, *n* = 521; one center from Shandong Province, *n* = 666) of China were reviewed. These three regions represent the middle, southern, and northern regions of China, and all patients underwent surgery in the comprehensive treatment group of spine surgery in each center. We divided the patients into three age groups, 50–59, 60–69 and > 70 years, to better analyze the status of osteoporosis among these patients of different ages. The inclusion criteria were as follows: (1) patients aged ≥ 50 years who underwent spine surgery. Exclusion criteria: (1) non-Asian race; (2) patients who were not examined using DXA within three months before surgery in each department; (3) patients who did not have the chest radiograph and spine radiograph necessary to evaluate VFs (T4-L4). This retrospective study was approved by the local ethics committee of each medical center involved. Since the study employed a retrospective design, informed consent was not required. All study methods were carried out in accordance with the relevant guidelines and regulations.

### BMD evaluation

BMD was measured in all patients from the four centers using GE Lunar DXA scanners (Prodigy or iDXA; GE Healthcare, Waukesha, WI, USA). DXA was performed at both the lumbar spine (L1-L4) and hips (femoral neck and total hip). The minimum T-value of BMD was adopted.

### Visual Radiography Assessment (VRA)

VRA was applied in the spine from T4-L4. All radiographic data were sent to one center. Two specifically trained doctors separately evaluated and compared the spine radiographs while being blinded to all data concerning the patients. If the conclusions did not match, a consensus was reached by discussion between the doctors. Kappa coefficients for intra- and interobserver agreement were 0.873 and 0.753, respectively (*P* < 0.001). VRA was performed on chest radiographs and spine radiographs, which were necessarily performed before surgery. For VFs related to trauma, metastatic tumors, tuberculosis, infection, and congenital deformity, we asked for a detailed medical history of fragility VFs, and VRA was applied to the non-lesion area to avoid interference from non-OP VFs in the lesion area. According to Genant’s semiquantitative grade classification [13], the VFs were defined as an at least 20 % reduction in the vertebral height.

### Diagnosis of osteoporosis

OP was diagnosed based on the World Health Organization criteria, and the lowest T-score in the lumbar spine or hips was applied to define normal (T > −1), osteopenia (− 1 ≥ T > − 2.5), or osteoporosis (T ≤ − 2.5) [14]. In addition, OP was diagnosed if a fragility VF was present in the absence of other metabolic bone diseases, independent of the T-score value. Fragility VFs are caused by low-level trauma, which can be compared to the force falling from a standing position or less and would not cause a fracture in healthy bone [15]. The detailed medical history of fragility VFs and the VRA of vertebrae from T4-L4 were collected to identify fragility VFs.

### Statistical analysis

IBM SPSS version 26 (Chicago, IL, USA) was used for statistical analyses. Based on the assumed OP prevalence of 48.9 % in females and 27.1 % in males [16], the sample size was estimated at the 5 % level of significance with the corresponding absolute error. The minimum estimated sample size in the present study was 186 for females and 479 for males. Continuous variables are presented as means ± SD, and categorical variables are reported as percentages (%). McNemar’s chi-squared test was used to compare the prevalence of OP between different groups. When inter- and intraobserver bias were considered, the Kappa statistic was used to evaluate the level of agreement in VRA results. *P* values < 0.05 were considered statistically significant.

## Results

According to the inclusion and exclusion criteria, 1,245 (67.1 %) patients were included in the final analysis, of which 493 patients were from Guangdong Province, 303 patients were from Chongqing municipality, and 449 patients were from Shandong Province. Among the 1,245 patients, the ratio of females (678) to males (567) was 1 to 0.84.

All enrolled patients had BMD data for the spine (L1-L4) and hips (femoral neck and total hip), and the lowest T-score was applied. Based on the T-score, 358 (52.8 %) cases of OP and 269 (39.7 %) cases of osteopenia were detected in females. When considering different age groups of females, the prevalence of OP was 28.8 %, 61.9 %, 75.7 % in the age groups of 50–59, 60–69, and > 70 years, respectively (*P* < 0.001). For males, 106 (18.7 %) cases of OP and 265 (46.7 %) cases of osteopenia were detected, and the prevalence of OP was 15.9 %, 17.1 %, and 33.8 % in the age groups of 50–59, 60–69, and > 70 years, respectively (*P* = 0.002). After removing the patients with osteoporotic VFs, 1,050 people were analyzed, including 543 females and 507 males. Based on the T-score, the prevalence of OP was 22.1 %, 59.2 %, 69.7 % in the 50–59, 60–69, and > 70 year age groups in females (*P* < 0.001). The age-standardized prevalence of OP in males in the 50–59, 60–69, and > 70 year age groups was 13.0 %, 13.2 %, and 26.8 %, respectively (*P* = 0.023). The incidence of OP in females showed an age-related increase (*P* < 0.001), but this trend was not observed in males between the 50–59 and 60–69 year age groups (*P* = 0.713). According to the T-score, the incidence of OP after excluding the cases of osteoporotic VFs was lower than the total, however, no significant difference was observed between the sexes (females, 52.8 % vs. 47.3 %, *P* = 0.057; males, 18.7 % vs. 14.6 %, *P* = 0.073) (Tables 1 and 2).

**Table 1:** Characteristics of study population

Variables	Total	50-59	60-69	≥70	*P*
Female					
n of DXA	678	232	339	107	
Age (years)	62.67±8.02	54.80±2.78	63.56±2.81	76.92±5.08	
n of osteoporosis by DXA (n %)	358(52.8%)	67(28.8%)	210(61.9%)	81(75.7%)	<0.001^*^
n of osteopenia by DXA (n %)	269(39.7%)	138(59.5%)	113(33.3%)	18(16.8%)	<0.001^*^
n of patients with fragility VFs in osteoporosis (n %)	221(61.7%)	32(47.8%)	135(64.3%)	54(66.7%)	0.031^*^
n of patients with fragility VFs in osteopenia (n %)	83(30.9%)	41(29.7%)	38(38.1%)	4(22.2%)	0.210
n of patients with fragility VFs in normal BMD (n %)	6(11.8%)	3(11.1%)	3(18.75%)	0	
n of osteoporosis by DXA and VRA (n %)	447(65.9%)	111(47.8%)	251(74.1%)	85(79.4%)	<0.001^*^
Male					
n of DXA	567	233	263	71	
Age (years)	61.72±8.04	54.07±2.87	64.46±2.59	76.65±4.66	
n of osteoporosis by DXA (n %)	106(18.7%)	37(15.9%)	45(17.1%)	24(33.8%)	0.002^*^
n of osteopenia by DXA (n %)	265(46.7%)	97(41.6%)	137(52.1%)	31(43.7%)	0.057
n of patients with fragility VFs in osteoporosis (n %)	50(47.2%)	13(35.1%)	17(37.8%)	20(83.3%)	<0.001^*^
n of patients with fragility VFs in osteopenia (n %)	81(30.6%)	31(32.0%)	40(29.2%)	10(32.3%)	0.895
n of patients with fragility VFs in normal BMD (n %)	43(21.9%)	21(21.2%)	18(22.2%)	4(25.0%)	
n of osteoporosis by DXA and VRA (n %)	230(40.6%)	89(38.2%)	103(39.2%)	38(53.5%)	0.058

*DXA* dual-energy X-ray absorptiometry, *BMD* bone mineral density, *VRA* visual radiography assessment, *VF* vertebral fracture

^*^P<0.05, comparisons among the 50-59, 60-69, and over 70 age groups

**Table 2:** Characteristics of study population except the primary diagnosis of osteoporotic vertebral fracture

Variables	Total	50-59	60-69	≥70	*P*
Female					
n of DXA	543	195	272	76	
Age (years)	62.24±7.77	54.82±2.74	63.56±2.81	76.92±5.01	
n of osteoporosis by DXA (n %)	257(47.3%)	43(22.1%)	161(59.2%)	53(69.7%)	<0.001*
n of osteopenia by DXA (n %)	238(43.8%)	127(65.1%)	96(35.3%)	15(19.7%)	<0.001*
n of patients with fragility VFs in osteoporosis (n %)	120(46.7%)	8(18.6%)	86(53.4%)	26(49.1%)	<0.001*
n of patients with fragility VFs in osteopenia (n %)	52(21.8%)	30(23.6%)	21(21.9%)	1(6.7%)	0.168
n of patients with fragility VFs in normal BMD (n %)	3(6.3%)	1(4.0%)	2(13.3%)	0	
n of osteoporosis by DXA and VRA (n %)	312(57.5%)	74(37.9%)	184(67.6%)	57(71.1%)	<0.001*
Male					
n of DXA	507	216	235	56	
Age (years)	61.47±7.81	54.06±2.88	64.47±2.57	76.13±4.56	
n of osteoporosis by DXA (n %)	74(14.6%)	28(13.0%)	31(13.2%)	15(26.8%)	0.023*
n of osteopenia by DXA (n %)	240(47.3%)	89(41.2%)	125(53.2%)	26(46.4%)	0.039*
n of patients with fragility VFs in osteoporosis (n %)	18(24.3%)	4(14.3%)	3(9.8%)	11(73.3%)	<0.001*
n of patients with fragility VFs in osteopenia (n %)	56(23.3%)	23(25.8%)	28(22.4%)	5(19.2%)	0.828
n of patients with fragility VFs in normal BMD (n %)	40(20.7%)	21(21.2%)	16(20.3%)	3(20.0%)	
n of osteoporosis by DXA and VRA (n %)	170(33.5%)	72(33.3%)	75(31.9%)	23(41.1%)	0.426

*DXA* dual-energy X-ray absorptiometry, *BMD* bone mineral density, *VRA* visual radiography assessment, *VF* vertebral fracture

^*^P<0.05, comparisons among the 50-59, 60-69, and over 70 age groups

OP can be diagnosed in the clinic without a BMD measurement if a low-energy fragility VF occurs in the vertebra. Among the 1,245 patients, 484 fragility VFs were detected, of which 310 occurred in females and 174 occurred in males. The prevalence of OP increased considerably when VRA was added to the BMD classification. Among the 678 female patients, the total prevalence of OP was 65.9 % (447/678), and the prevalence was 47.8 %, 74.1 %, and 79.4 % in the 50–59, 60–69, and > 70 year age groups, respectively (*P* < 0.001). The relative number increased by 24.9 %, and the absolute number increased from 52.8 to 65.9 % when VRA was added to the BMD classification. After excluding the female patients whose primary diagnosis was osteoporotic VFs, the total prevalence of OP was 57.5 % (312/543), and it was 22.1 %, 59.2 %, and 69.7 % in the 50–59, 60–69, and > 70 year age groups, respectively (*P* < 0.001). The relative number and the absolute number increased by 21.4 and 10.1 %, respectively, which was similar to the total data from females. For the 567 male patients, the total prevalence of OP sharply increased from 18.7 to 40.6 % with the additional diagnosis based on VRA. Considering different age groups, the prevalence was 38.2 %, 39.2 %, and 53.5 % in the 50–59, 60–69, and > 70 year age groups, respectively (*P* = 0.058). After excluding the males whose primary diagnosis was osteoporotic VFs, the total prevalence of OP was 33.5 %, 33.3 % in the 50–59 year age group, 31.9 % in the 60–69 year age group, and 41.1 % in the > 70 year age group (*P* = 0.426). For all males and males without a primary diagnosis of osteoporotic VFs, the relative number increased to 117.0 and 129.7 %, respectively, and the absolute values increased by 21.9 and 18.9 %, respectively. Although the prevalence of OP in females was much higher than that in males (*P* < 0.001), no significant difference in the fragility VF rate was observed in the non-OP (osteopenia or normal BMD) group when the comparisons were performed between different age groups of females and males (females vs. males: 50–59 years, *P* = 0.977; 60–69 years, *P* = 0.302; >70 years, *P* = 0.172). Regardless of whether the patients who were primarily diagnosed with osteoporotic VFs were excluded, we did not observe a significant difference in the prevalence of non-OP fragility VFs within different age groups of females and males (total patients: females, *P* = 0.210; males, P = 0.895; patients excluding those with osteoporotic VFs: females, *P* = 0.168; males, *P* = 0.828) (Tables 1 and 2).

**Fig. 1:**
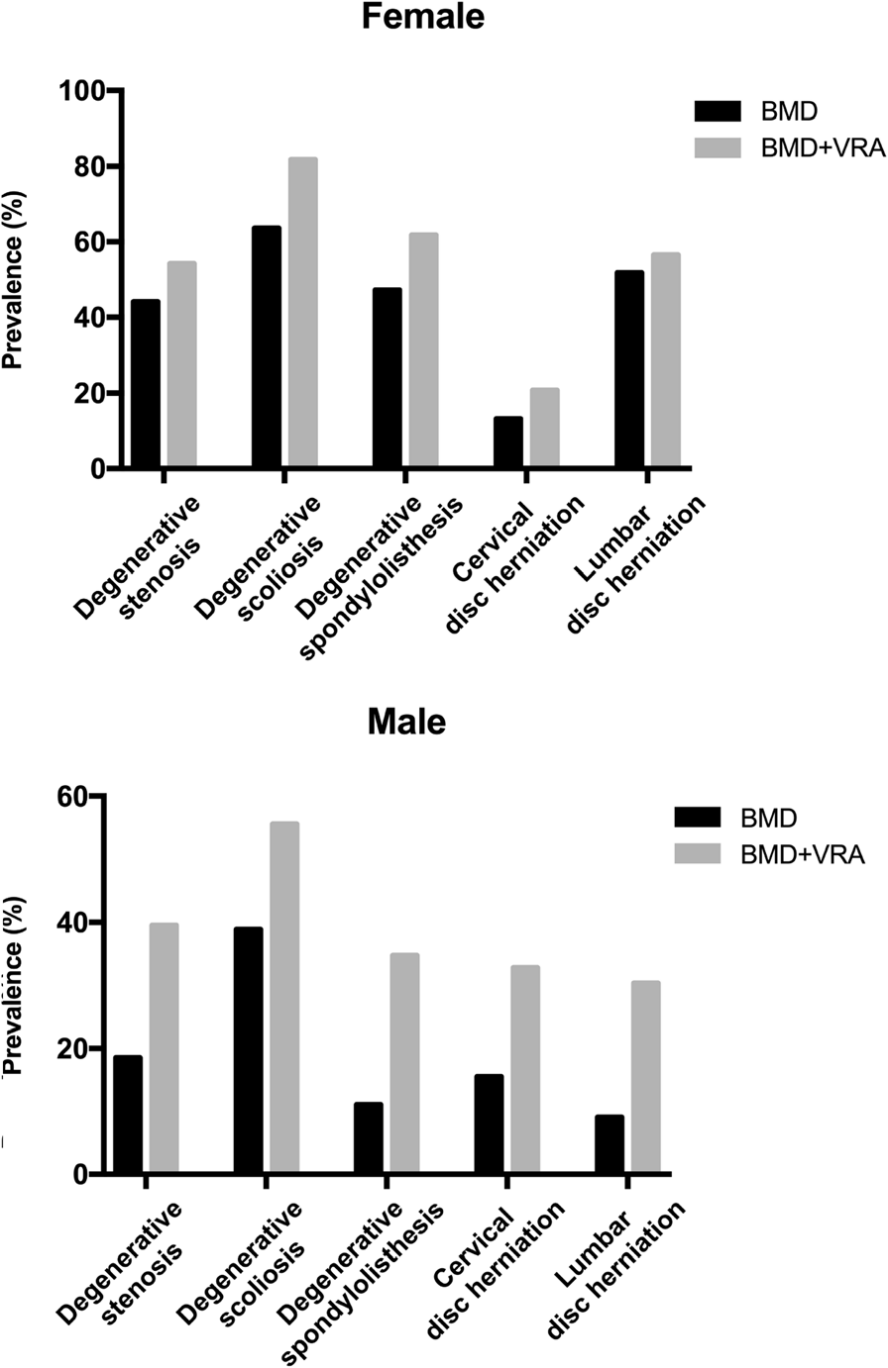
The prevalence of osteoporosis among different spine degenerative diseases in the female and the male

The prevalence of OP showed a remarkable difference among patients with different spine degenerative diseases (SDDs). Whether using DXA alone or in combination with VRA, the prevalence of OP in females whose primary diagnosis was an SDD was significantly higher than that in males (44.0 % vs. 15.0 %, *P* < 0.001; 54.2 % vs. 35.8 %, *P* <0.001) (Table 3). The prevalence of OP diagnosed by BMD in patients with degenerative scoliosis was higher than that in patients with the remaining degenerative diseases among both females and males (females: 63.6 % vs. 42.4 %, *P* = 0.018; males: 38.9 % vs. 13.8 %, *P* = 0.004); however, when VRA was combined with BMD for classification, this difference only remained in females (females: 81.8 % vs. 52.0 %, *P* = 0.001; males: 55.6 % vs. 34.8 %,* P* = 0.073). Females with degenerative scoliosis who were not diagnosed with OP by BMD had the highest incidence rate of VFs compared to the remaining females (50.0 % vs. 16.7 %, *P* < 0.001), but no significant difference was identified in males (27.3 % vs. 24.3 %, *P* = 0.733). The incidence rate of OP in females diagnosed with lumbar disc herniation (51.8 %) and degenerative spondylolisthesis (47.2 %) by BMD was followed by females with degenerative scoliosis (63.6 %); however, degenerative spondylolisthesis was more common than lumbar disc herniation after adding a diagnosis based on VRA (61.8 % vs. 56.5 %). Unlike females, the prevalence of OP in males diagnosed with degenerative spinal stenosis (18.5 %) and cervical disc herniation (15.5 %) by BMD was lower than that diagnosed with degenerative scoliosis (38.9 %), but the cervical disc herniation diagnosis was changed to degenerative spondylolisthesis after combining DXA and VRA. The prevalence of OP in patients with many other SDDs is shown in Table 3; Fig. 1.

**Table 3:** Prevalence of osteoporosis in different spine degenerative diseases

Variables	Number of patients (n)	Osteoporosis diagnosed by DXA (n, n %)	Osteoporosis diagnosed by DXA and VRA (n, n %)	Patients with fragility VFs in non-osteoporosis (n, n %)
Female				
Total	439	193(44.0%)	238(54.2%)	45(18.3%)
Degenerative stenosis	179	79(44.1%)	97(54.2%)	18(18.0%)
Degenerative scoliosis	33	21(63.6%)	27(81.8%)	6(50.0%)
Degenerative spondylolisthesis	89	42(47.2%)	55(61.8%)	13(27.7%)
Cervical disc herniation	53	7(13.2%)	11(20.8%)	4(8.7%)
Lumbar disc herniation	85	44 (51.8%)	48(56.5%)	4(9.8%)
Male				
Total	366	55(15.0%)	131(35.8%)	76(24.4%)
Degenerative stenosis	119	22(18.5%)	47(39.5%)	25(25.8%)
Degenerative scoliosis	18	7(38.9%)	10(55.6%)	3(27.3%)
Degenerative spondylolisthesis	72	8(11.1%)	25(34.7%)	17(26.6%)
Cervical disc herniation	58	9(15.5%)	19(32.8%)	10(20.4%)
Lumbar disc herniation	99	9(9.1%)	30(30.3%)	21(23.3%)

*DXA* dual-energy X-ray absorptiometry, *VRA* visual radiography assessment, *VF* vertebral fracture

## Discussion

The study included data from 3 different regions of China and aimed to explore the prevalence of OP in patients older than 50 years who underwent spine surgery. According to the present study, the prevalence of OP diagnosed by BMD in patients undergoing spine surgery in China was 37.3 % (464/1245), including 52.8 and 18.7 % in females and males, respectively. The prevalence of OP varies by country in patients scheduled for spine surgery, ranging from 40 to 60 % in females and 10–20 % in males [16–19]. Compared to other countries, our results were relatively high. The prevalence of OP diagnosed by BMD in females in our study was similar to that in another study conducted in China, but the prevalence in males in our study was lower [20]. However, this Chinese study included a more limited and smaller sample compared to our study.

Whether the diagnosis based on VRA was included, the prevalence of OP drastically increased with age in females (*P* < 0.001) but not in males. Several studies in China showed that the prevalence of OP diagnosed by BMD in females over 50 years of age ranged from 9 to 23 %, 26–45 %, and 41–67 % in the 50–59, 60–69, and > 70 year age groups, while in males, it was 2–11 %, 6–18 % and 14-32.5 %, respectively [1, 21–23]. We observed an apparently higher prevalence of OP diagnosed by BMD in patients who underwent spine than that in a general population, especially in females. Osteoporotic VF is extremely common in the aging Chinese population and comprises a large portion of the patients in our study; thus, so the prevalence of OP will be increased. However, even if patients with osteoporotic VFs were excluded, the prevalence of OP in females and males only decreased to 47.3 and 17.6 %, respectively, which still far exceeded the values observed for general female (29.13 %) and male (6.46 %) populations [2].

Osteoporotic VF, namely, fragility VF, is the most common osteoporotic fracture and directly affects the diagnosis of OP, fracture risk prediction, and clinical management. The latest osteoporotic guidelines proposed by the American Association of Clinical Endocrinologists (AACE) in 2020 suggested that the fracture risk assessment tool (FRAX) should be highly valued, and a personal history of fragility fractures, including radiographic VFs, was the key contributing factor [15]. In our study, fragility VFs detected by VRA were observed in 213 (17.1 %) non-OP patients, of which 89 were females and 124 were males. Compared with the use of BMD alone, the relative diagnostic efficiency of OP increased to 24.9 %, and the absolute prevalence increased from 52.8 to 65.9 % in all females when OP was defined by both VRA and BMD. Namely, 13.1 % of females (89/678) with OP would be ignored if BMD was the only evaluation performed. In addition, 17.7 % of females (120/678) with OP diagnosed by BMD worsened into a severe prognosis due to the detection of fragility VFs. However, this phenomenon was more serious in male patients. The combined diagnosis (BMD and VRA) increased the relative number of patients with OP by 117 %, and the absolute value increased from 18.7 to 40.6 % in all males. Related data from patients without osteoporotic VFs were similar to the total cohort. Similar studies in other countries have also reported the high prevalence of fragility VFs from T4-L4. Approximately 20 years ago, a study involving 482 postmenopausal women searched for methods to prevent and treat OP. Fragility VFs were detected in 18.3 % of these women, and the prevalence of OP increased from 10.6 to 26.1 % based on total hip BMD and from 25.1 to 38.6 % based on spine BMD [24]. In a prospective study conducted in Denmark, 585 males aged 60–74 years in the general population were referred to assess BMD, of which the prevalence of OP diagnosed by BMD increased from 10.2 to 14.8 % when the vertebral fracture assessment (VFA) was added to the classification [25]. Another study focused on Danish females also reported an increase in the number of patients with OP by 9.79 % when VFA and BMD were applied simultaneously [26]. A survey from northern India showed that the prevalence of VF in healthy community-dwelling men was 24.5 % in the 60–70 year age group and 38.4 % in the 71–80 year age group [27]. Another recent study focused on postmenopausal women in China found that 62.4 % of patients were diagnosed with OP and the number diagnosed with severe OP increased significantly by 17.2 % based on the combination of VFA and BMD [28]. Comparing the aforementioned studies with ours, we found significantly higher prevalence rates of OP and fragility VFs in Asians than those in Caucasians, and the previously unknown fragility VFs were extremely common in males. In the present study, 26.8 % of males with osteopenia or normal BMD had fragility VFs, which was remarkably higher than the value reported in other countries. One reason is that the race of the patients analyzed in these studies was different. In the authors’ opinion, the other reason is that VFs assessed in other studies were based on the DXA scan; however, we applied radiography to diagnose VFs, which would be a more careful and accurate approach. Finally, China is experiencing unprecedented aging. Some recent studies from China have proposed that the incidence rate of VFs increases significantly with age in elderly Chinese females and males [29, 30], but interestingly, no significant difference in the prevalence of fragility VFs was detected in females and males without OP among different age groups in the present study. Therefore, all age groups, rather than only the elderly, should receive the same attention when we evaluate the bone status of patients aged > 50 years who are undergoing spine surgery.

Fragility VF is a potentially severe complication of OP that is closely related to persistent back pain, spine deformity, and increased mortality. VF might also be associated with many postoperative complications, such as constipation, stroke, pneumonia, urinary tract infection, arrhythmia, loss of height, anxiety for future fractures, and a two to three times higher mortality risk, particularly in patients with symptomatic VFs [31, 32]. Patients who underwent spine surgery due to osteoporotic VFs comprised a large proportion of the cases, and although fragility VFs caused by low-energy injuries in the elderly can be directly diagnosed with OP, a large number of surgeons still ignore the bone loss in these patients. This negligence is more serious in patients whose primary diagnosis is not osteoporotic VFs. A questionnaire provided to orthopedic surgeons and neurosurgeons reported that 40 % of them did not check BMD when treating patients with low-energy spine fractures. Even worse, only 44 % of surgeons checked BMD before a spine fusion operation, compared with 22 % before a noninstrumented fusion [33]. Neglecting the preoperative BMD examination may cause serious consequences. A multicenter, multiracial study showed that patients with OP undergoing degenerative cervical spine surgery were more likely to undergo revision surgery, have longer hospitalizations and have higher hospitalization costs than their counterparts without OP [34]. Therefore, spine surgeons should pay more attention to OP in all patients aged > 50 years who are undergoing surgery, which will detect more patients with OP and help to adjust the operation strategy in a timely manner. The combination of BMD and VRA appears to be a better approach for detecting undiagnosed OP than either assessment alone.

Although many studies have examined the relationship between spinal degeneration and OP, few studies have focused on the distribution of OP among patients with SDDs. A study in South Korea performed over a decade ago investigated the distribution of OP diagnosed by BMD among patients aged > 50 years with different SDDs requiring spine surgery. In their study, the top three SDDs were degenerative stenosis (42.9 %), degenerative spondylolisthesis (38.3 %), and lumbar disc herniation (30.7 %) in females, and degenerative spondylolisthesis (14.8 %), cervical disc herniation (11.8 %) and lumbar disc herniation (7.9 %) in males [16]. Compared with their results, our study is slightly different. In the present study, the top three incidence rates of OP diagnosed by BMD among different types of SDDs were degenerative scoliosis (63.6 %), lumbar disc herniation (51.8 %), and degenerative spondylolisthesis (47.2 %) in females, and the corresponding SDDs in males were degenerative scoliosis (38.9 %), degenerative spinal stenosis (18.5 %) and cervical disc herniation (15.5 %). Patients with scoliosis were not included in the study from South Korea, and their incidence rate of OP among patients with different SDDs was lower than the values reported in our study. The rapidly expanding aging phenomenon in China may explain the difference. Another recent study in China analyzed the BMD and the Hounsfield units of 479 patients aged > 50 years who required lumbar fusion for lumbar degenerative diseases and found that the prevalence of OP in patients with scoliosis was 56.5 %, which was higher than the prevalence in the remaining patients [20]. In our study, whether using BMD alone or combined with BMD and VRA, the prevalence of OP in patients with degenerative scoliosis was always higher than that in the remaining patients, consistent with the results of the Chinese study described above. However, in the discussion of the prevalence of OP among patients with different SDDs, this Chinese study did not distinguish between sexes, which is different from our study. The prevalence of OP was significantly different between females and males, as is the distribution of SDDs. In the present study, we provide better estimates of the specific situations of females and males. In addition, compared to this single-center study from northern China, the patients in the present study resided in the northern, middle, and southern regions of China, which might make the results more accurate. At present, many studies have attempted to clarify the relationship between OP and SDDs, but a clear conclusion has not been reached. Generally, the compression of the nerve root and the deformation of vertebrae caused by SDDs lead to a decrease in activities and cause falls, resulting in OP and fragility VFs. At the same time, OP leads to microfractures and then decreases the height of the motion segment and the stability of facet joints, which ultimately aggravates SDDs [35].

### Limitations

Our study has some limitations. First, we assessed fragility VFs based on the visual semiquantitative method developed by Genant et al., which is one of the most widely used and simplest methods in observational studies and clinical trials, but it is not the gold standard method. Some different semiquantitative approaches have been developed to identify VFs, such as instant VFA applied using a DXA scanner and conventional semiquantitative radiography using the six-marker point method, but they share the same sensitivity and specificity as VRA [36]. Furthermore, because our study was a retrospective analysis, VRA was performed on patients’ chest and spine radiographs captured in preparation for surgery, which would result in a certain error due to blurring of the spine in part of the chest. Therefore, prospective randomized trials are needed to confirm the findings. In addition, we included the patients with trauma, metastatic tumors, tuberculosis, infection, and congenital deformity in present study. For these special patients, DXA were performed at both lumbar spine (L1-L4) and hips (femoral neck and total hip) and the minimum T-value was adopted. We also asked them for a detailed medical history of fragility VFs, and the VRA was applied to the non-lesion area to avoid the interference of non-OP VFs in lesion area. Few other similar studies included these specific patients, but many studies have shown that tumor and inflammation are closely related to the occurrence of OP [37, 38]. Finally, current guidelines in China for senile OP recommend a BMD measurement in asymptomatic females and males aged > 65 and 70 years, respectively, and standard spine radiography or VFA is indicated for females aged > 70 years and males aged > 80 years with a T-score < -1. In principle, all patients requiring spine surgery in our study were recommended to evaluate BMD voluntarily, and VRA was performed based on chest X-ray and the lateral spine radiograph, which must be performed before surgery; thus, the patients did not experience an additional burden.

## Conclusions

OP is extremely common among patients aged > 50 years who are undergoing spine surgery. For this special population, a general assessment of BMD and VRA prior to spine surgery should be considered in patients over 50 years of age, especially patients whose primary diagnosis is degenerative scoliosis. The combination of BMD and VRA can identify more unknown cases of OP and result in fewer complications of spine surgery.

## Data Availability

All data that support the results of this study are available from the corresponding author on request.
